# Preparation and characterization of a novel magnetic nano adsorbent for removal of metal ions

**DOI:** 10.1371/journal.pone.0329686

**Published:** 2025-08-01

**Authors:** Mutairah S. Alshammari, Marwa M. El-Tyieb, Mariam E. Fawzy, Eida S. Al-Farraj, Hussein M. Ahmed

**Affiliations:** 1 Department of Chemistry, College of Science, Jouf University, Sakaka, Aljouf, KSA; 2 Housing and Building Research Center (HBRC), Sanitary and Environmental Institute. Dokki, Egypt, Giza, Egypt; 3 Water Pollution Research Department, National Research Centre, Dokki, Giza, Egypt; 4 Department of Chemistry, College of Science, Imam Mohammad Ibn Saud Islamic University (IMSIU), Riyadh City, KSA; University of Szeged, HUNGARY

## Abstract

As a result of global urbanization and industrialization, heavy metals are one of the hazardous contaminants facing the world. The adsorption process using agricultural wastes can achieve one of the sustainable development goals for wastewater treatment and resource recovery. Moringa and tea extracts were utilized to synthesize iron nanoparticles for the treatment of aqueous solutions containing heavy metal ions (Cu^2+^, Pb^2+^, Se^2+^, Zn^2+^, and Cr^6+^). This method offers a sustainable substitute for conventional chemical wastewater treatment methods. Furthermore, the use of magnetic iron nanoparticles reduces the need for extra separation processes by making it simple to separate the adsorbent from the treated waste using a magnetic field. Various techniques were employed to characterize the prepared nanoparticles, such Fourier-transform infrared spectroscopy (FT-IR), energy dispersive x-ray spectroscopy (EDX), scanning electron microscopy (SEM), x-ray diffraction (XRD), and x-ray fluorescence (XRF). The XRD analysis confirmed the crystalline phase of alpha-FeNPs in the synthesized nanoparticles. The EDX analysis verified the presence of oxygen and iron in the nanoparticles, indicating that the iron was in an oxide form. This study aimed to investigate the removal of heavy metals using nano-magnetic composites of moringa (FeNPs-M) and tea (FeNPs-T). To assess the effectiveness of the FeNPs-M several parameters were tested, including pH, contact time, initial concentration, and nanoparticle dosage. The results indicated that the efficiency of FeNPs-M was significantly higher than that of FeNPs-T for the removal of heavy metals from synthetic solutions, achieving removal efficiency are 96.5% 99.71%, 96.73%, 93.16%, and 91.83% of Cu^2+^, Pb^2+^, Se^2+^, Zn^2+^, and Cr^6+^, respectively, when using FeNPs-M, while the removal efficiency are 96.36%, 93.40%, 79.83%, 78.6%, and 77.77% of Cu^2+^, Pb^2+^, Se^2+^, Zn^2+^, and Cr^6+^, respectively, 25 °C, with a contact time of 45 min, a pH of 3.0, concentration 3.0 mg/L, a sorbent dose of 0.8 g/L, and 200 rpm at 25 °C.

## 1. Introduction

Water pollution is one of the most important issues facing our current era. Among the pollutants found in wastewater are heavy metals. The sources of wastewater vary in terms of the type of water or the source of pollution as well as the degree of pollution [1]. The pollutants from water pollution have a detrimental effect on health, particularly due to the presence of heavy metals in the food chain Pengcheng Guo et al 2025 [2]. When heavy metals enter the human body through the water, they can cause serious diseases such as cancer, Alzheimer’s, and birth defects in fetuses [1,3]. Heavy metals that can be found in wastewater include chromium, copper, lead, nickel, zinc, and cadmium [4–7]. They are toxic, non-biodegradable reported to Mahmoud, S.A et al 2025 [8], and their presence in water has several impacts on aquatic life [9–12]. One of these heavy metals is zinc. The amount of zinc in water may increase due to industrial sources or toxic waste sites Peng, Y et al 2024 [13]. Consuming even a small amount of zinc can result in a loss of appetite. Hence, it was necessary to find a way to remove these heavy metals as well as to treat and dispose of the pollutants in wastewater.

There are several treatment methods available, including wetlands, natural or chemical coagulants, adsorption, and ion exchange [14,15].

Nanoparticles can be prepared using various methods, including physical and chemical processes for water treatment. Various synthesis techniques such as the sol-gel method, hydrothermal synthesis, chemical vapor deposition (CVD), and electrochemical processes each come with their own limitations. Electrochemical methods, for instance, often face challenges such as broad size distribution of the products, high operational costs, and difficulties in precisely controlling deposition parameters. The sol-gel technique, while widely used, is highly sensitive to moisture, difficult to scale up for industrial applications, and typically involves multiple, time-intensive steps. Additionally, it can lead to dimensional and volumetric changes throughout the process. CVD methods, though effective, require elevated processing temperatures and involve costly equipment and maintenance. The process is also complex, time-consuming, and poses safety risks due to the use of toxic gases and high temperatures. Hydrothermal synthesis mainly results in nanoparticles that crystallize in a metastable cubic phase, which generally lacks ferroelectric properties. Moreover, using water as the reaction medium may introduce hydroxyl (OH) groups into the iron nanoparticle (FeNP) structure, leading to structural defects, altered chemical stoichiometry, and ultimately, lower product quality as reported by Piao Xu et al 2012 [16]. An alternative method, known as “green synthesis,” offers a cost-effective and eco-friendly approach to nanoparticle synthesis as reported by Al Shammari et al 2023 [17]. This method utilizes natural materials such as plants and microorganisms, making it an attractive option for preparation. These natural materials contain substances that aid in the biological reduction of nanoparticles, the presence of vitamins, proteins, amino acids, phenolic acids, and alkaloids [18] are considered as effective molecules with unique characteristics. Among the substances present in plants and microorganisms are phenolic acids that contain carboxyl and hydroxyl groups capable of binding metals, making them powerful antioxidants. The conversion of metal ions into metal nanoparticles may be attributed to active hydrogen [19]. One of the types of nanoparticles produced through green synthesis is magnetic iron particles. These particles are widely utilized in water treatment due to their exceptional physical and chemical properties. They stand out from other compounds because of their larger specific surface area and a higher proportion of atoms on their surface in comparison to their nuclei. Magnetic separation techniques offer various advantages, including high efficiency and low cost, attributed to the larger specific surface area and lower internal diffusion resistance of these materials compared to other nano-metals used in the separation process [20–22]. Various forms of iron oxides, such as Fe₃O₄ (magnetite), Fe₂O₃ (hematite), and FeOOH, have been extensively explored for their ability to degrade organic pollutants and reduce toxicity through enhanced photocatalytic activity. Among these, magnetite (Fe₃O₄) and hematite (Fe₂O₃) are naturally occurring iron oxides with distinct characteristics. Magnetite contains a higher proportion of iron and exhibits inherent magnetic properties, whereas hematite becomes magnetic only when heated. In terms of appearance, magnetite is typically black, while hematite can appear in a range of colors depending on its specific form and composition reported by Khedr M et al [23]. Many plants are used in the process of synthesizing nano iron particles, like moringa. It contains antioxidants [10,24] with a short chain length of low molecular weight of cationic polyelectrolyte that contribute to its effectiveness [25]. Moringa nanoparticles are potential adsorbents for removing pollutants [26]. Studies have demonstrated that moringa nanoparticles are capable of effectively removing a variety of substances, such as metals and organic pollutants [21].

Tea black residues can be used in wastewater treatment due to its cost-effectiveness. It enhances the treatment efficiency. The cell wall of tea black residues is made up of cellulose, lignin, and carbohydrates, all of which contain hydroxyl groups. These functional groups, which constitute approximately one-third of the dry mass of tea residues, have shown significant potential for the removal of heavy metals from wastewater [1]. The integration of iron magnetic nanoparticles by chemical co-precipitation method as a potential adsorbent was synthetized by combining with natural extracts (tea or moringa). This combination brings out the best of both components, improving the adsorption capacity and selectivity for heavy metal ions. It also provides an environmentally eco-friendly alternative to traditional chemical wastewater treatment processes. In addition, the use of magnetic iron nanoparticles allows the adsorbent to be easily separated from the treated waste using a magnetic field, thus reducing additional separation steps. The aim of this study is to develop a suitable, environmentally friendly, and cost-effective method for synthesizing iron nanoparticles (FeNPs) using natural extracts from moringa and tea (M, T). These FeNPs will be evaluated for their effectiveness in removing heavy metals specifically Cu² ⁺ , Pb² ⁺ , Se² ⁺ , Zn² ⁺ , and Cr⁶⁺ from synthetic aqueous solutions. Through a series of experiments, the study also seeks to identify optimal conditions that allow for the reuse of these nanoparticles in the treatment of industrial and sewage wastewater.

## 2. Materials and methods

### 2.1. Moringa and tea extracts synthesis

**Fig 1:**
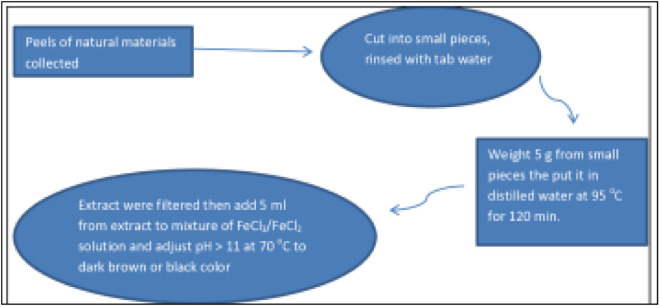
Schematic diagram of the iron nanoparticles preparation in presence extract of moringa and tea.

The moringa extract solution was prepared by collecting a group of moringa leaves from the moringa tree, then cutting them into small pieces, then placing 5.0 g of moringa leaves in a liter of distilled water, and heating for 2 h at 95 °C, then filtering the extracts through filter paper (No. 1) and storing the extracts in the refrigerator at 4 °C [27]. The same procedure and steps were used to prepare the tea extract. Fig 1 is a schematic diagram for the preparation of iron nanoparticles from tea, and moringa materials using green synthesis method. The extracts are stored at a temperature of 4°C for a maximum of three months, as this is the longest period during which the same results and quality can be maintained for the prepared and synthesized nanomaterials. Prolonged storage at inappropriate temperatures leads to changes in the composition of the extract and promotes the growth of microorganisms, which can negatively affect its efficiency and composition. The tea used is black tea of the Lipton brand, sourced from Egypt.

### 2.2. Green preparation of Fe_3_O_4_ nanoparticles and preparation of metal ions solutions

Tea or moringa extracts were mixed with a solution containing FeCl_3_.6H_2_O and FeCl_2_.4H_2_O to synthetize magnetic iron nanoparticles. Mixing 5 mL of the extract with 1.0 L of FeCl_3_/FeCl_2_ solution, the extracts were converted into Fe_3_O_4_ nanoparticles. This process was carried out at 70 °C and mixed at 10,000 rpm for 2 h, and NaOH (1.0 M, MERCK, 99.9%) solution was added dropwise at the same time to neutralize and adjust the pH (pH > 11) [28]. The color of the solution changed from orange to black throughout the process of nanoparticle synthesis, and the appearance of a black precipitate indicated the formation of FeNPs [29,30]. The iron nanoparticles were collected and dried in a dry oven at 50 °C for 48 h after being separated by centrifugation at 10,000 rpm [31]. Stock solutions of one g/L of each metal (Cu^2+^, Pb^2+^, Se^2+^, Zn^2+^, and Cr^6+^) dissolved in HNO_3_ were used to create synthetic heavy metal solutions. (HNO_3_) and sodium hydroxide (NaOH) solutions of 0.1 M each were made using deionized water.

### 2.3 The Fe_3_O_4_ nanoparticles morphological examination

Several tools were used to characterize the magnetite nanoparticles (FeNPs) that had been synthesized. To analyze synthesized FeNPs as a function of time, for instance, a UV–visible spectrophotometer (T-70 spectrophotometer, PG Instruments Limited is a spectrometer with wavelength range 190 nm, 1100 nm, spectral resolution 0.5 to 5 nm at the Housing and Building National Research Centre, Chemistry Lab) was utilized on a regular basis with a resolution of 0.5 nm in the wavelength range of 190–340 nm. Using CuK α radiation from 40 kV/30 mA and a 2θ range of 20–70°, X-ray diffraction (XRD 6100, Shimadzu, Tokyo, Japan) was used to conduct a crystallographic analysis of FeNPs. The elemental analysis was done using EDX (EDX 720, Shimadzu, Tokyo, Japan), and chemical functional group identification on FeNPs was determined using FT-IR (FT-IR 8400S, Shimadzu, Tokyo, Japan) in the 400–4000 cm^-1^ spectrum range. Ahmed et al., 2023 [32] explained the instruments used in this study.

### 2.4 Adsorption records

The adsorption study was carried out by combining 0.1–1.0 g of **Fe**_**3**_**O**_**4**_ nanoparticles with one liter of 0.5–3.0 mg/L heavy metal solution (Cu^2+^, Pb^2+^, Se^2+^, Zn^2+^, and Cr^6+^) at 200 rpm for 30 min. To guarantee that the results were of high quality, these experiments were repeated three times to achieve results with high accuracy and also to ensure the efficiency of the materials and devices used. An atomic absorption spectrometer (AAS) equipped with a graphite furnace (ICE 3000 AAS, provided by Thermo Fisher Scientific American) was used to assess the heavy metals according to APHA [33]. The operating conditions (initial metal concentration, contact time, and pH) were carried out according to Ahmed et al., 2023 [32]. Batch studies were conducted to examine the impact of adsorbent dose on the performance of heavy metals removal, as carried out according to Sowmiya, K. et al., 2019 [35]. A synthetic aqueous solution of heavy metals was combined with the adsorbent at various levels (0.5, 1.0, 2.0, 3.0, 4.0, and 5.0 mg/L), at the same dose of 0.8 g and contact time of 45 min. Adjust pH values using (HCl 1.0 N, MERCK, 37%, and NAOH 1.0 N, MERCK, 99.9%).

### 2.5 Isotherm study

Adsorption isotherms are important to describe the adsorption mechanism of a solute on adsorbent surface thus aid in optimizing the design of a specific adsorption process. In the present study, the equilibrium data obtained for heavy metals removal on FeNPs-M and FeNPs-T was tested with two isotherm models Langmuir and Freundlich isotherm equations. Isotherm coefficients and correlation coefficients (*R*^*2*^) were computed from linearized equations of these isotherms in Microsoft Excel.

#### 2.5.1 Langmuir isotherm approach.

Equilibrium studies that provide the capacity of the adsorbent and adsorbate are described by adsorption isotherms, which are typically the ratio between the amount adsorbed and that which was left in solution in the equilibrium phase at a constant temperature [34]. The Langmuir model is based on the following assumptions: there is no adsorbate molecule migration in the surface plane, the adsorption energy is constant, and maximal adsorption occurs when there is a saturated monolayer of solute molecules on the adsorbent surface. Monolayer sorption is driven by physical processes, according to the Langmuir isotherm model. The following is the Langmuir isotherm equation:


$$qe=\frac{C.Kqmax}{1+KL}\mathrm{\backslash ]}$$ (1)

$$\frac{Ce}{qe}=\frac{1}{qmax\;\;.\;KL}+\frac{Ce}{qmax}\mathrm{\backslash ]}$$ (2)

$$RL=\frac{1}{1+Co.KL}\mathrm{\backslash ]}$$ (3)

where q_e_ is the amount of metals adsorbed on a certain dose of adsorbent (mg/g), C_e_ is the solution’s equilibrium level (mg/L), and q_max_ is the highest amount of metals needed to produce a monolayer (mg/g). The Langmuir constants are denoted by q_max_ and K. The linear plot of C_e_/q_e_ vs C_e_ can be used to calculate the values of q_m_ and K [36].

#### 5.2 The Freundlich isotherm approach.

According to the Freundlich isotherm approach, the empirical connection that represents the adsorption of solutes from a liquid surface to a solid surface involves several sites with different adsorption energies. K_F_ and n, which represent the system’s adsorption intensity and capacity, respectively, are its properties. The ability of the Freundlich model to fit the experimental data was examined. Using the plot of log C_e_ vs. log q_e_, the intercept value of K_F_ and the slope of n were determined for this case. When the surface is heterogeneous and the absorption is multilayered and bonded to specific places on the surface, the Freundlich isotherms emerge.


$$\log qe=\log KF+\frac{1}{n}\log Ce\;\mathrm{\backslash ]}$$ (4)

where 1/n = Intensity parameter and K_F_ is the Freundlich equilibrium constant (mg/g). C_e_ is the adsorbate’s equilibrium concentration, and q_e_ is the amount of solute adsorbed [37–39]. Freundlich approach is represented by the equation, which plots log q_e_ vs. log C_e_ linearly (4) [14]. The Freundlich formulation yields the constants K_F_ and 1/n in a linear form. In a multilayer covering, the Freundlich isotherm model postulates non-ideal adsorption on heterogeneous surfaces. It implies that weaker binding sites are occupied after stronger binding sites, the binding strength declines with increasing site occupation.

### 2.6 Kinetic models

The pseudo-first and pseudo-second-order kinetic models for each adsorbent could be described as follows:

#### 2.6.1 Pseudo first-order kinetics.

The pseudo-first-order kinetics is represented by Equation 5, which is the Lagergren model:


$$Log\;\left(q_{eq}-\;q_{t}\right)=\;logq_{eq}-\;K_{1}.\;t/2.303$$ (5)

Here, K_1_ is the pseudo-first-order constant (min^-1^) and qt is the adsorbed dosage of metal ions (mg/g) in t time (min). It is possible to calculate q_eq_ and K_1_ separately using the linear and angular constants of the log graphic (q_eq_ – q_t_) in the function of time

#### 2.6.2 Second-order kinetics.

Contrasting the values for q_eq_ that were obtained experimentally and determined using Equation 6:


$$t/q_{t}=\;1/K_{2}{q_{eq}}^{2}+\;t/\;$$ (6)

where q_eq_ is determined using an angular coefficient, and K_2_ is the pseudo-second-order constant (g/mg. min) that is derived by computing a linear coefficient [35,14]. To evaluating the goodness of fit for non-linear kinetic models (pseudo-first-order, and pseudo-second) in adsorption and biodegradation processes, error analysis is essential such as Sum of Squared Errors (SSE).


$$SSE=\sum_{i=1}^{n}(qexp-qcal).^{2}$$

Where q_e, exp_, is the experimental adsorption capacity and q_e, cal_ is the calculated value from the model. Lower SSE values indicate a better fit of the model to the experimental data, and highly sensitive to large deviations.

## 3. Results

### 3.1 The adsorption approach of iron nanoparticles

In a few seconds, the color of the solution changes from translucent brown to black, which indicates the formulation of iron nanoparticles and validates the development of FeNPs-M and FeNPs-T nanoparticles [31]. The black coloration of the synthesized iron particles indicates the formation of magnetite-type iron nanoparticles (Fe₃O₄) reported by Sadia et al 2016 [40]. This characteristic not only confirms the presence of higher iron content but also suggests natural magnetic properties and high efficiency, as reported by Wehua et al 2022 [41].

#### 3.1.1 The FT-IR investigation.

**Fig 2:**
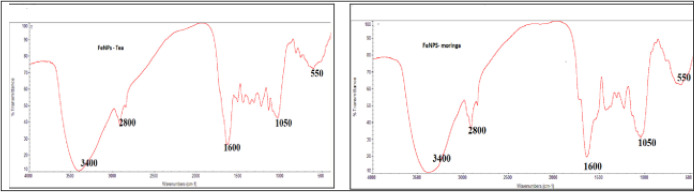
The FT-IR spectrum of (a) FeNPs-M and (b) FeNPs-T investigations.

The molecules that may be in charge of reducing metal precursor ions and acting as a stabilizing agent for nanoparticles were identified using FT-IR investigation. The FT-IR spectra of moringa and tea extracts were recorded in the range of 400–4000 cm ⁻ ¹, as illustrated in Fig 2(a,b). A prominent band observed at 3420 cm ⁻ ¹ in both spectra corresponds to the O–H stretching vibration of phenolic groups, which are believed to play a key role in the reduction of iron ions reported by Al Shammari et al 2023 [17]. Furthermore, many characteristic peaks of the plant extracts are also present in the spectra of the synthesized Fe₃O₄ nanoparticles, indicating that these bioactive compounds contribute to the stabilization and capping of the nanoparticles [30]. The C-H atoms’ methyl group stretching produces broad peaks at 2822 cm^-1^. The carbonyl groups (C = O) caused a peak at 1695 cm^-1^, which altered the OH group’s stretching and vibration frequency to 3269 cm^-1^. The emergence of a new frequency peak at >700 cm^-1^ in the spectra of Fe_3_O_4_ nanoparticles is explained by the vibrations of the iron oxide’s Fe-O bonds [31]. The presence of a C = O peak around 1695 cm ⁻ ¹ could also arise from residual organic solvents used during the extraction or synthesis process, especially if those solvents contain carbonyl functional groups.

#### 3.1.2 The SEM investigations of FeNPs-M and FeNPs-T.

**Fig 3:**
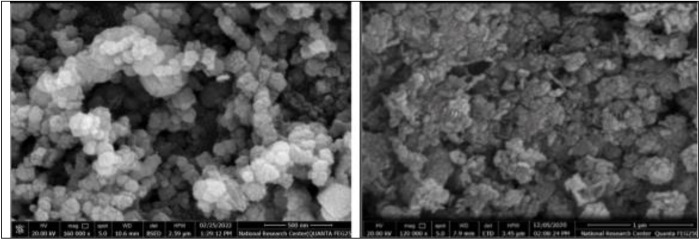
The SEM of (a) FeNPs-M and (b) FeNPs-T investigation.

For morphological characterization, the SEM micrographs of the produced magnetic FeNPs-M nanoparticles were carried out. The SEM was used to examine the FeNPs-M and FeNPs-T materials’ shape, size, and surface. The SEM images for FeNPs-M and FeNPs-T nanoparticles are shown in Fig 3(a,b). It demonstrates the high adsorption capacity and superior porosity of the FeNPs-M. When comparing the FeNPs-T SEM study to the one in Fig 3a, it can be concluded that the structure is uniform. White spots on the adsorbent’s surface in Fig 3b have an irregular aggregation structure and scattered distribution. Moreover, Fig 3b demonstrates the creation of nanoscale FeNPs-M and FeNPs-T particles, with diameters of 50.8 and 80.2 nm, respectively [42]. Table 1 also displays the summarizing particle size and elemental compositions of FeNPs-M and FeNPs-T. Fig 3 exhibits a degree of particle aggregation, which is commonly observed in magnetic nanoparticles due to van der Waals forces and magnetic dipole–dipole interactions. Such aggregation can decrease the effective surface area, thereby potentially reducing adsorption efficiency. To address this, ultra-sonication was performed for (15–20) min prior to all adsorption experiments to enhance nanoparticle dispersion in solution. This procedural detail will be clearly stated in the Methods section.

**Table 1:** The summarizing particle size and elemental compositions of FeNPs-M and FeNPs-T.

Iron oxide nanoparticle	FeNPs-T	FeNPs-M
Fe_3_O_4_%	40.86	46.62
Particle size (nm)	50.8	80.2

#### 3.1.3 The XRD f FeNPs-M and FeNPs-T.

**Fig 4:**
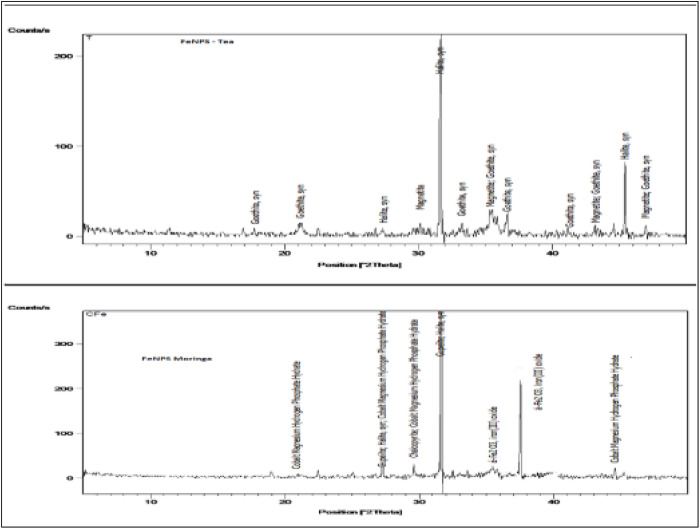
The XRD profile of (a) FeNPs-M and (b) FeNPs-T.

The XRD was generated to look into the existence of nanoparticles on the surfaces of the tea and moringa leaves. The exact FeNPs-M and FeNPs-T nanoparticle peaks occur between 2θ = (10° and 60°). A notable peak at 2 values of 46° is seen in the approach of this nanoparticle for the crystalline structures of FeNPs-M and FeNPs-T. The structure of the produced iron nanoparticles was determined using the XRD method. The XRD profile of the generated adsorbent employing Cu kα radiation (λ = 1.5 Å) is shown in Fig 4(a,b) in the angle range of 2θ. Using this method, carbon (C) has been located at the 24.7° peaks. The peak at an angle of 45.5° offers more proof that there are Fe_3_O_4_ particles in the adsorbent framework. In general, the XRD investigation supported the successful coating of Fe_3_O_4_ particles on the surfaces of the tea and moringa. Diffraction peaks with 2θ values of 46° were found in the observed XRD profile depicted in Fig 4(a,b). The results of the X-ray diffraction (XRD) analysis of the iron nanoparticles (FeNPs-M) and (FeNPs-T) confirm that the iron molecules are formed in the crystalline phase of iron particles (Fe_3_O_4_) [30,31,42]. The peak observed at approximately 46° was attributed to Fe₃O₄ (magnetite) based on the JCPDS reference card #89–3854. Additional characteristic peaks at 30.2°, 35.5°, 43.1°, 53.4°, 57.0°, and 62.6° were also matched with standard diffraction patterns, further confirming the crystalline structure of Fe₃O₄. We will ensure this reference is cited explicitly in the revised manuscript reported to Elisa Bertolucci et al 2015 [44].

#### 3.1.4 The FeNPs-M and FeNPs-T XRF pattern.

Table 2 shows the XRF profile of iron oxide nanoparticles derived from tea and moringa. Table 2 also displays the proportion of Fe_3_O_4_ composite in FeNPs-M and FeNPs-T. The Fe_3_O_4_ percentage in FeNPs-M (46.62%) is higher than that in FeNPs-T (40.86%), indicating FeNPs-M higher efficiency. In the context of Fe₃O₄-based materials, a high Loss on Ignition (LOI) value may indicate the presence of moisture. The LOI method involves heating the sample to high temperatures. Moisture content, in particular, can significantly impact LOI values if samples are not properly dried beforehand, leading to overestimated mass loss and potentially underestimated Fe₃O₄ content. Therefore, ensuring proper drying and solvent removal is critical to obtaining reliable FT-IR and compositional data [30].

**Table 2:** The XRF of Fe_3_O_4_ nanoparticle FeNPs-M and FeNPs-T.

Iron oxide nanoparticle	Fe-metal	FeNPs-T	FeNPs-M
Fe_3_O_4_%	47.28	40.86	46.62
Na_2_O %	11.7	19.8	22.1
MnO %	0.43	0.33	0.46
CaO %	0.09	0.47	0.27
SiO_2_%	0.28	0.38	0.36
Al_2_O_3_%	0.06	0.11	0.07
K_2_O %	0.00	0.03	0.07
SO_3_^2-^ %	0.11	0.13	0.00
Cr_2_O_3_%	0.04	0.01	0.04
MgO %	0.00	0.07	0.00
Cl^-^ %	10.1	15.6	14.9
LOI %	29.9	22.2	15.1
Total %	99.99	99.99	99.99

### 3.2 The yield of the produced nanoparticles

Table 3 depicts the XRF profile of iron oxide nanoparticles derived from tea and moringa. Table 3 also displays the proportion of Fe_3_O_4_ composite in FeNPs-M and FeNPs-T. The Fe₃O₄ percentage reported in Table 1 is based on weight percentage (wt%), as is standard in most EDX analyses [30].

**Table 3:** The Yield of Fe_3_O_4_ nanoparticle FeNPs-M and FeNPs-T.

Iron oxide nanoparticle	Weight (g)
Fe-metal	7.625
FeNPs-M	38.235
FeNPs-T	12.899

### 3.3 Influence of the pH value

**Fig 5:**
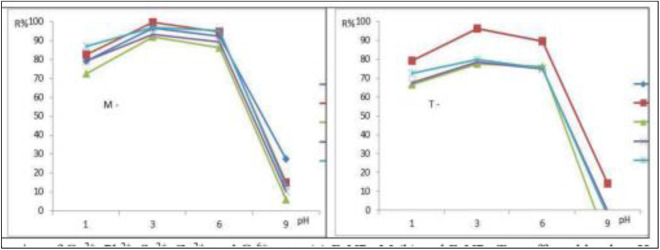
Adsorption of Cu^2^^+^, Pb^2^^+^, Se^2^^+^, Zn^2^^+^, and Cr^6^^+^, onto (a) FeNPs-M (b) and FeNPs-T as affected by the pH value at the optimum operating conditions.

Fig 5(a,b) illustrate how the pH value interferes with the absorption of Cu^2+^, Pb^2+^, Se^2+^, Zn^2+^, and Cr^6+^. The pH of the solution alters the surface charge, degree of ionization, and separated target and active groups on the composite [42]. The composite’s surface charge, level of ionization, and segregated target and active groups are all affected by the solution’s pH [43]. The results indicate that the removal efficiency of Cu² ⁺ , Pb² ⁺ , Se² ⁺ , Zn² ⁺ , and Cr⁶ ⁺ decreases as the pH increases. This trend can be explained by changes in the surface charge of the iron nanoparticles (FeNPs). At lower pH levels, the FeNPs surface carries a positive charge (approximately +20 mV at pH 3.0), which enhances its electrostatic attraction to negatively charged species such as Pb₂O₃² ⁻ , SeO₂² ⁻ , Cr₂O₇² ⁻ , ZnO₂² ⁻ , and CuO₂²⁻ [12,43]. However, as the pH rises, the surface charge of the FeNPs becomes increasingly negative, reducing their affinity for these anionic metal species and thereby lowering the overall adsorption efficiency reported by Alvaro et al 2019 [45]. Subsequently increases the electrostatic attraction of the Cu^2+^, Pb^2+^, Se^2+^, Zn^2+^, and Cr^6+^, ions to the adsorbent as a result. This causes the adsorbed Cu^2+^, Pb^2+^, Se^2+^, Zn^2+^, and Cr^6+^, species to be released from the FeNPs surface. Moreover, as Cu^2+^, Pb^2+^, Se^2+^, Zn^2+^, and Cr^6+^, can easily oxidize Fe_3_O_4_ particles to Fe^2+^ at pH 6.0, this facilitates the adsorption of these metals. Since FeNPs-M achieved the highest levels of Cu^2+^, Pb^2+^, Se^2+^, Zn^2+^, and Cr^6+^, adsorption at pH 3.0 was 99.71%, 96.5%, 90.22%, 91.83%, and 96.73, respectively.

It follows that the reduction process under acidic circumstances increases the effectiveness of Cu^2+^, Pb^2+^, Se^2+^, Zn^2+^, and Cr^6+^, removal. The ideal pH was chosen as this one. This finding is in good agreement with past studies. According to a related study, pH 3.0 was the optimal pH for eliminating Cu^2+^, Pb^2+^, Se^2+^, Zn^2+^, and Cr^6+^ [43]. Inversely, iron becomes insoluble when pH rises and generates ferrous hydroxide, which builds up on the surface of the iron and obstructs mass transfer. Because the surfaces of metal ions and nanoparticles are negatively charged in alkaline circumstances, this phenomenon produces repulsive interactions between the ions and insoluble forms of iron [46]. These active groups may be segregated at alkaline solutions, where they could occupy FeNPs-M and FeNPs-T composites [42,47]. The pH of experiment > 11.0 in synthesis FeNPs but in adsorption study of heavy metals removal (optimum condition) pH 3.0, Fig 5 clears the relation between the removal efficiency (R%) with pH values). The potential reduction of Cr⁶⁺ to Cr³⁺ during adsorption was considered, as this redox behavior can significantly influence both the adsorption mechanism and efficiency process. Cr³⁺ and Cr⁶ ⁺ have different chemical properties, including charge, size, and affinity for binding sites on the adsorbent. Cr³ ⁺ , being a cation, tends to interact differently with the surface functional groups compared to anionic Cr⁶ ⁺ species (CrO₄²⁻ or HCrO₄⁻), potentially altering the nature and strength of the adsorption. The Cr⁶ ⁺ is known to be a strong oxidizing agent and can be reduced to the more stable Cr³ ⁺ form, especially in the presence of reducing agents commonly found in plant extracts or other bio-adsorbents. The Cr⁶ ⁺ is reduced to Cr³⁺ during the process, some portion of the chromium removed from solution may not be due solely to physical adsorption, but rather to a chemical transformation. This can lead to an overestimation of adsorption efficiency if only total chromium removal is measured without distinguishing between oxidation states.

Although Cr⁶ ⁺ , Pb² ⁺ , and Cu² ⁺ have comparable ionic radii, the lower removal efficiency of Cr⁶ ⁺ is primarily attributed to differences in speciation and pH dependent behavior, rather than ionic size alone. Under typical environmental or experimental pH conditions (pH 4–6), Cr⁶ ⁺ exists predominantly as oxyanions, such as CrO₄²⁻ (chromate) or HCrO₄⁻ (hydrogen chromate). In contrast, Pb²⁺ and Cu² ⁺ remain as positively charged cations. Since most adsorbents—especially iron oxide-based materials like Fe₃O₄ develop a negatively charged surface at neutral to slightly acidic pH, they favor the adsorption of cationic species (Pb² ⁺ , Cu²⁺) through electrostatic attraction and chemisorption via coordination with surface functional groups such as –OH and –COOH. On the other hand, the negatively charged Cr⁶ ⁺ oxyanions experience electrostatic repulsion, leading to reduced adsorption efficiency. Furthermore, effective removal of Cr⁶ ⁺ often depends on its reduction to Cr³ ⁺ , or anion exchange mechanisms, which are less favorable or require specific conditions that may not be present during standard adsorption experiments.

The stability of Fe₃O₄ under low pH conditions can be maintained through several experimental and material-based factors. Short contact times and controlled temperatures during the adsorption process help minimize prolonged exposure to acidic environments, thereby reducing the likelihood of Fe₃O₄ dissolution. Additionally, surface modifications such as organic or inorganic coatings applied to FeNPs-M and FeNPs-T may offer protection against acid-induced degradation, enhancing the chemical resilience of the nanoparticles. Furthermore, the presence of complexing agents or adsorbed metal ions can contribute to the formation of a protective surface layer, which limits further interaction with protons (H⁺). Thus, although low pH conditions promote proton availability, the combination of optimized experimental parameters and surface stabilization mechanisms likely prevented significant Fe₃O₄ dissolution during adsorption at pH 3.

### 3.4. Influence of contact time

**Fig 6:**
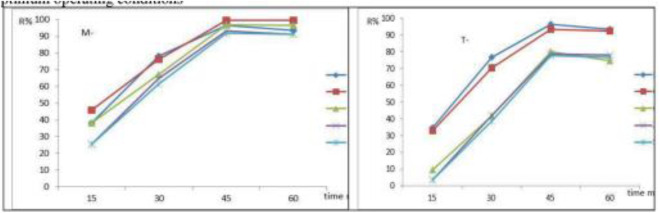
Adsorption of Cu^2^^+^, Pb^2^^+^, Se^2^^+^, Zn^2^^+^, and Cr^6^^+^, onto (a) FeNPs-M and (b) FeNPs-T as affected by the contact time at the optimum operating conditions.

Fig 6 (a,b) shows how contact time affects the adsorption of heavy metals under the given conditions: A dose of 0.3 g/L of the adsorbent solution, a contact time of 0–60 min, and an optimum pH of 3.0 ± 0.1. The effectiveness of Cu^2+^, Pb^2+^, Se^2+^, Zn^2+^ and Cr^6+^, adsorption rose significantly up to 45 min before beginning to decline. As illustrated in Fig 6 (a,b). Numerous empty active sites on the adsorbent surface may be the cause of the sudden increase in adsorption performance. The amount of Cu^2+^, Pb^2+^, Se^2+^, Zn^2+^, and Cr^6+^ ions that are accessible to the active sites on the adsorbent surface is limited by lengthening the contact duration. The adsorption capacity is lowered as a result [42]. This effect was investigated in related investigations with different adsorbents. The results showed that 45 min was the best duration for employing magnetic nanoparticles to remove metals [42,48]. Fig 6 clears the relation between the removal efficiency (R%) with times effect values). All tests were at room temperature, the decrease in adsorption efficiency after 45 minutes, as shown in Fig 6 may be attributed to either surface saturation depending on the adsorption dynamics. As contact time increases, the availability of Cu² ⁺ , Pb² ⁺ , Se² ⁻ , Zn² ⁺ , and Cr⁶ ⁺ ions for binding to the active sites on the adsorbent surface becomes limited, likely due to the progressive occupation of adsorption sites and the system approaching equilibrium.

### 3.5. Influence of adsorbent dose

**Fig 7:**
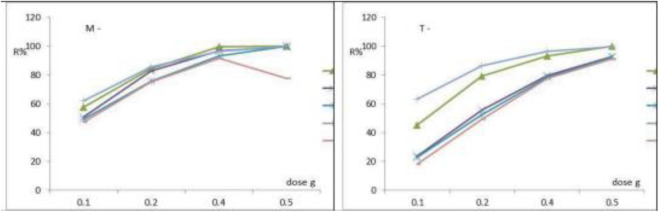
Influence of the mass of adsorbent (a) FeNPs-M and (b) FeNPs-T on removal of heavy metals at the optimum operating conditions.

We investigated how FeNPs-M and FeNPs-T magnetic nanoparticle levels (0.1–0.5 g/L) affected the removal of the under-consideration heavy metals. How well nanoparticles can adsorb contaminants depends on the quantity of those particles. The removal effectiveness rose as the dosage of magnetic particles increased, as shown in Fig 7(a,b). Iron nanoparticles often reduce metal ions as a surface function. This shows that the active site available on the nano-composite is decreasing with higher sorbent dosages [42,48,49]. The effects of different concentrations of FeNPs-M and FeNPs-T on the adsorption capacity and efficiency under optimal conditions (pH = 3, t = 45 min, and 200 rpm) are displayed in Fig 7(a,b). It can be shown that the removal efficiency increased from 62.23%, 57.73%, 51%, 49.13%, and 47.13% to 96.5% 99.71%, 96.73%, 93.16%, and 91.83% for 3.0 mg/L of Cu^2+^, Pb^2+^, Se^2+^, Zn^2+^, and Cr^6+^, respectively, when using FeNPs-M adsorbent. Also, the removal efficiency increased from 63.42%, 42.26%, 23.566%, 22.23%, and 18.13% to 96.36%, 93.40%, 79.83%, 78.6%, and 77.77% for 3.0 mg/L of Cu^2+^, Pb^2+^, Se^2+^, Zn^2+^, and Cr^6+^, respectively, when using FeNPs-T. The increased availability of active sites on FeNPs-M and FeNPs-T, which can result in the adsorption of ions, is connected to the increase in adsorption efficiency. Previous research showed that increasing the dosage of various adsorbents increased the removal of Cu^2+^, Pb^2+^, Se^2+^, Zn^2+^, and Cr^6+^. However, a decrease in adsorption capacity with an increase in adsorbent dosage is most likely due to the adsorption capacity per gram may decrease due to overlapping or aggregation effects at high dosages [50,51]. The adsorbent dose of 0.8 g/L was selected as the optimal concentration based on adsorption capacity and removal efficiency observed during batch experiments. Beyond this dose, the increase in removal efficiency became marginal, indicating a plateau likely due to saturation of available adsorption sites and a reduced adsorbate to adsorbent ratio. Increasing the dose from 0.1 to 0.8 g/L significantly improved ions removal efficiency, achieving over 90%. Doses above 0.8 g/L resulted in minimal improvement (<5%) in removal efficiency while increasing material costs, processing complexity, and the potential for particle agglomeration. Thus, 0.8 g/L offers the best balance between adsorption performance and economic practicality for potential scale-up.

### 3.6. Influence of initial heavy metals concentration

**Fig 8:**
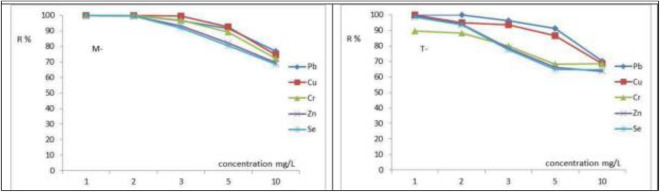
Influence of starting heavy metals level on removal efficiency using (a) FeNPs-M and (b) FeNPs-T at optimum operating conditions.

Fig 8(a,b) shows the influence of starting levels (1.0, 2.0, 3.0, 5.0, and 10 mg/L) of Cu^2+^, Pb^2+^, Se^2+^, Zn^2+^, and Cr^6+^ on the efficiency of the adsorption process. When increasing the starting levels of Cu^2+^, Pb^2+^, Se^2+^, Zn^2+^, and Cr^6+^ from 1.0 to 10 mg/L, the percentage of adsorption capacity of FeNPs-M decreased from 100%,100%, 100%, 100%, and 100% to, 74.4%, 76.85%, 68.5%, 69.5%, and 72.2%. The adsorption capacity of FeNPs-T decreased from 100%, 99.85%, 89.4%, 99.38%, and 98.6% to 70.2%, 68.45%, 68.5%, 63.5%, and 64.4% for Cu^2+^, Pb^2+^, Se^2+^, Zn^2+^, and Cr^6+^, respectively. The aforementioned results seem to be mostly caused by the adsorbent’s limited number of active sites on its surface [21,48,50]. The concentrations of Ca² ⁺ , Mg² ⁺ , Na ⁺ , and K⁺ ions in both the synthetic solution and real wastewater were very low; therefore, these ions did not significantly affect the removal efficiency due to minimal competition for active sites. These results highlight the adsorbent’s selectivity and will inform further optimization efforts for treatment of real wastewater.

### 3.7 Adsorption isotherm

**Fig 9:**
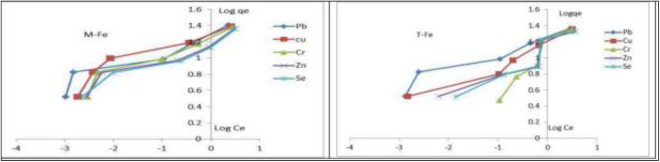
Freundlich isotherms for heavy metals adsorption on (a) FeNPs-M and (b) FeNPs-T.

Table 4 displays the results for the correlation coefficient (R^2^) of Isotherm models for adsorption of Cu^2+^, Pb^2+^, Se^2+^, Zn^2+^, and Cr^6+^on FeNPs-M and FeNPs-T at 25 ± 1°C. The Freundlich isotherm model clearly outperforms the Langmuir isotherm model in terms of (R^2^ > 0.99). This outcome shows that the Freundlich model and the experimental results have a good degree of agreement. Additionally, it appears from Fig 9(a,b) that the obtained data may be aligned with the Freundlich model. The Freundlich isotherm describes adsorption on heterogeneous surfaces and assumes that stronger binding sites are occupied first reported by Vigdorowitsch et al 2021 [53], and the Cu^2+^, Pb^2+^, Se^2+^, Zn^2+^, and Cr^6+^ are adsorbed on FeNPs-M and FeNPs-T in a multilayer fashion.

**Table 4:** The correlation coefficient (R^2^) of Isotherm models for adsorption of heavy metals on FeNPs-M and FeNPs-T.

Isotherm models	Nano composites	Pb	Cu	Cr	Zn	Se	Standard deviation (SD) values
R^2^ of Freundlich isotherm model	FeNPs-M	0.9456	0.9638	0.9277	0.9486	0.9463	0.012825
	FeNPs-T	0.9223	0.9401	0.9888	0.9407	0.8774	0.04008
R^2^ of isotherm model Langmuir	FeNPs-M	0.7562	0.7553	0.7592	0.9431	0.9126	0.094263
	FeNPs-T	0.9246	0.8293	0.9813	0.8219	0.8526	0.068791

Also, the values of R_L_ are seen to be between 0 and 1.0, showing that the Cu^2+^, Pb^2+^, Se^2+^, Zn^2+^, and Cr^6+^ ions have been successfully adsorbed on FeNPs-M and FeNPs-T. Based on the Langmuir model, the maximum uptake of FeNPs-M and FeNPs-T per mass unit was 25.61 and 22.83 mg/g, respectively, this is similar to what has been reached from several previous studies Kakavandi, B., et al 2014 [50]. Based on the results, the adsorption process appears to conform to both the Langmuir and Freundlich isotherm models, suggesting the involvement of monolayer adsorption as well as surface heterogeneity. The values of the Langmuir separation factor (R_L_) were observed to fall between 0 and 1.0, indicating that the adsorption of Cu² ⁺ , Pb² ⁺ , Se² ⁻ , Zn² ⁺ , and Cr⁶ ⁺ ions onto FeNPs-M and FeNPs-T was favorable. According to the Langmuir isotherm model, this implies that a significant portion of adsorption occurs on well-defined homogeneous sites. However, the excellent fit to the Freundlich model (R² > 0.99) indicates that the adsorption also takes place on a heterogeneous surface, where the active sites vary in energy and affinity. This heterogeneity leads to non-uniform adsorption behavior, often involving multilayer adsorption **or** variable binding strengths, which cannot be fully explained by the Langmuir model alone.

### 3.8 Kinetics of adsorption

**Fig 10:**
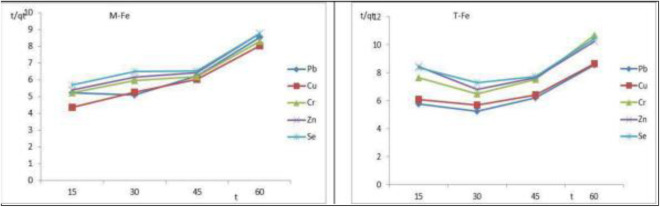
Kinetic models for heavy metals adsorption on (a) FeNPs-M and (b) FeNPs-T.

Table 5 lists the constant values for the kinetic models for the adsorption of heavy metals on FeNPs-M and FeNPs-T as well as the related regression (R^2^). The adsorption kinetics of Cu^2+^, Pb^2+^, Se^2+^, Zn^2+^, and Cr^6+^ on FeNPs-M were 0.9434, 0.9448, 0.8919, 0.9492, and 0.9465, respectively, and for FeNPs-T were 0.8198, 0.9408, 0.9562, 0.8608, and 0.9722. The curves shown in Fig 10 further support this finding (a, b). The study of data from the pseudo-second-order pattern leads to a conclusion that chemisorption regulates the adsorption of heavy metals onto FeNPs-M and FeNPs-T. Chemisorption is a form of adsorption characterized by the formation of chemical bonds between the adsorbent and the adsorbate. This process typically involves the sharing or transfer of electrons, leading to the creation of covalent bonds that result in strong and often irreversible interactions. The pseudo-second-order kinetic model is commonly used to describe chemisorption, as it assumes that the rate-limiting step involves electron sharing or exchange between the adsorbent and adsorbate. This model reflects the dependence of the adsorption rate on the availability of active sites and the chemical nature of the interaction reported by Piao et al 2012 [16]. Hence, it is clear that the pseudo-second-order model best describes the kinetics of heavy metal adsorption on FeNPs-M and FeNPs-T. This is consistent with earlier observations on the adsorption of heavy metals [50]. The adsorption process follows a pseudo-second-order kinetic model, which suggests that the rate-limiting step involves chemisorption, typically associated with electron sharing or exchange between the adsorbent and adsorbate, evidence supporting electron sharing (chemisorption) such as high correlation (R² > 0.99) with the pseudo-second-order model, indicating the dominance of chemical bonding over physical interactions, and FT-IR spectral shifts. Changes in functional group peak positions (–OH, –COOH, C = O) suggest the involvement of specific surface functional groups in bonding, consistent with electron donation/acceptance mechanisms. The q_exp_ of FeNPs-M and FeNPs-T per mass unit was 25.61 and 22.83 mg/g, respectively, and the q_e,cal_ in first order model was 7980 mg/g, but in second order model was 23.4 mg/g. Based on the error analysis using the SSE, the pseudo-second-order kinetic model exhibited the lowest SSE value among the evaluated models. This indicates a superior fit between the model-predicted and experimental data, confirming that the adsorption process follows pseudo-second-order kinetics, which suggests that chemisorption may be the rate-limiting step involving valence forces through sharing or exchange of electrons between adsorbent and adsorbate.

**Table 5:** The adsorption kinetic models’ parameters regarding the of heavy metals onto FeNPs-M and (b) FeNPs-T.

kinetic models	Pb	Cu	Cr	Zn	Se	SD
R^2^ of pseudo second-order	0.9448	0.9434	0.8198	0.9492	0.8919	0.055513
	0.9408	0.9465	0.9722	0.8608	0.9562	0.043314
R^2^ of pseudo first-order	0.8004	0.8159	0.8559	0.8171	0.8805	0.033106
	0.6765	0.8820	0.5038	0.5968	0.896	0.055513

### 3.9 Comparison and recycling study of the prepared adsorbents on removal of heavy metals

Table 6 shows the comparison of several studies on the removal of heavy metals using nano iron particles. Desorption experiments were conducted to evaluate the adsorbent’s reuse potential. 0.1 M HCl was used as the desorbing agent and demonstrated high efficiency in removing adsorbed metal ions. The adsorbent underwent five adsorption/desorption cycles. After each cycle, it was thoroughly washed, dried, and reused. The adsorption efficiency remained above 80% after three cycles, followed by a gradual decline in subsequent cycles, likely due to partial blockage of active sites or structural changes in the adsorbent. These results confirm the material’s promising reusability and cost-effectiveness for multiple treatment cycles.

**Table 6:** Represents different magnetic adsorbents for the removal of heavy metal from aqueous solutions.

Type of pollutant	Adsorbent type and dose	Efficiency	Reference
Pb(II)	Zero-valent iron nanoparticles, 2 g/L, pH 6.0, and room temperature	99%	[52]
Cu(II)	Fe₃O₄&chitosan, 0.3 g/L, pH 6.0, and 21 °C	94%	[54]
Cr(VI)	γ-Fe_3_O_4_/SiO₂, 0.5 g/L, pH 6.0, and 23 °C	92%	[55]
Se(IV)	Fe₃O₄-rGO(graphene), 0.5 g/L, pH 6.0, and room temperature	98%	[56]
Cu (II), Pb(II), Zn(II), Se (I\I) and Cr (VI)	FeNPs-M	96.5%, 99.71%, 96.73%, 93.16%, and 91.83%, respectively at 3g/L	Present study

## Conclusions

The FeNPs-M and FeNPs-T were utilized to remove heavy metals from the aqueous solutions using the prepared nano-composite (Fe_3_O_4_) as an adsorbent. Moringa nanoparticles that are magnetic have been synthesized by green methods. As a stabilizing agent for the manufacture of iron nanoparticles, moringa and tea extract were utilized. Several characterization methods, such as EDX, XRF, FT-IR, XRD, and SEM imaging, revealed that Fe_3_O_4_ nanoparticles tend to cluster into irregular shapes with an average particle size of 50.8 nm, confirming the development of Fe_3_O_4_ nanoparticles. The Fe_3_O_4_ nanoparticles were found to have a crystalline structure according to their XRF and XRD approaches. The outcomes demonstrated the great effectiveness of the synthetic adsorbent in the adsorption of heavy metals. The ideal adsorption conditions were attained at a temperature of 25°C, a contact time of 45 min, and an acidic pH 3.0. Furthermore, the results of the equilibrium and kinetic analyses showed that the Freundlich isotherm and pseudo-second-order kinetic models were followed by the adsorption of heavy metals. Consequently, FeNPs-M and FeNPs-T can be used as efficient adsorbents for the treatment of water and wastewater. Therefore, our hybrid treatment process sustains a sustainable, economic, and cost-effective approach for heavy metal remediation. Laboratory experiments play a crucial role in evaluating the performance of treatment materials under controlled conditions. To achieve this, treatment models are often applied to laboratory synthetic solutions that are free from the organic pollutants and complex contaminants typically present in industrial wastewater. This controlled approach allows for the precise monitoring of adsorption behavior. However, recognizing the importance of real-world applicability, further studies are currently underway using real industrial wastewater samples.

## Supporting information

S1 Fe NPs +HM + supporting data (Autosaved). (XLSX)

S2 Data. (DOCX)
