# Supplementary material for: Preparation and characterization of a novel magnetic nano adsorbent for removal of metal ions
Source: PLoS One. 2025 Aug 1;20(8):e0329686. doi: 10.1371/journal.pone.0329686 (PMC12316269; doi:10.1371/journal.pone.0329686)

| 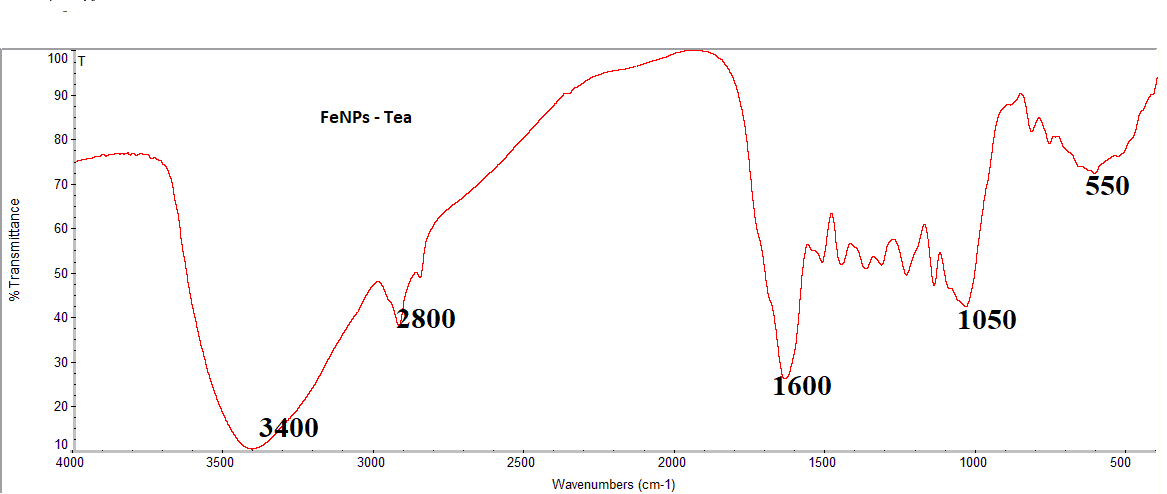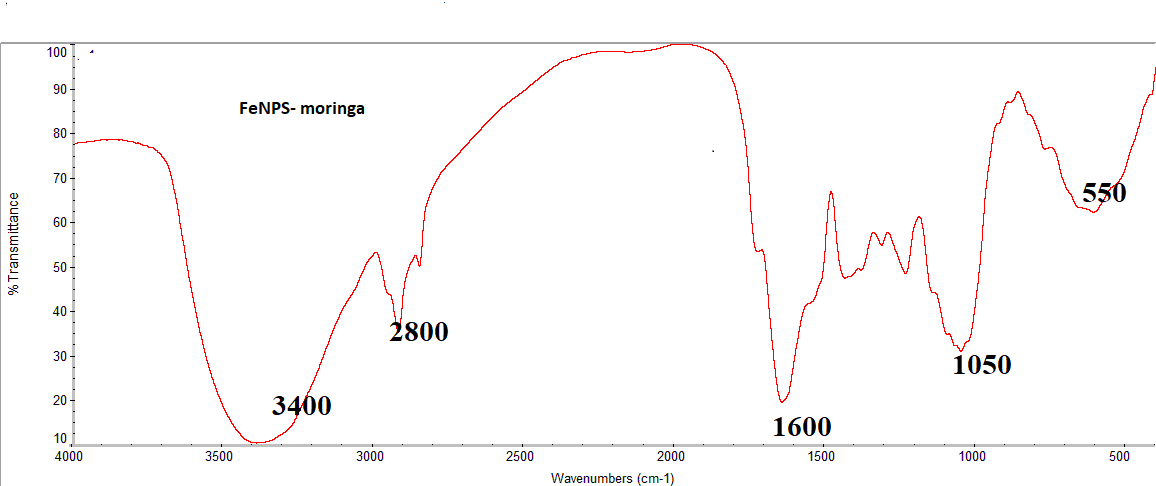 |  |
| --- | --- |

The FT-IR spectrum of (a) FeNPs-M and (b) FeNPs-T investigations


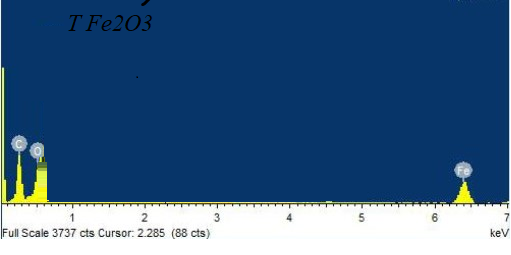

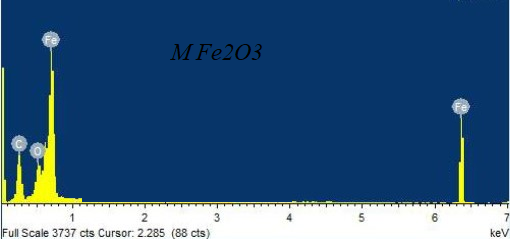


| **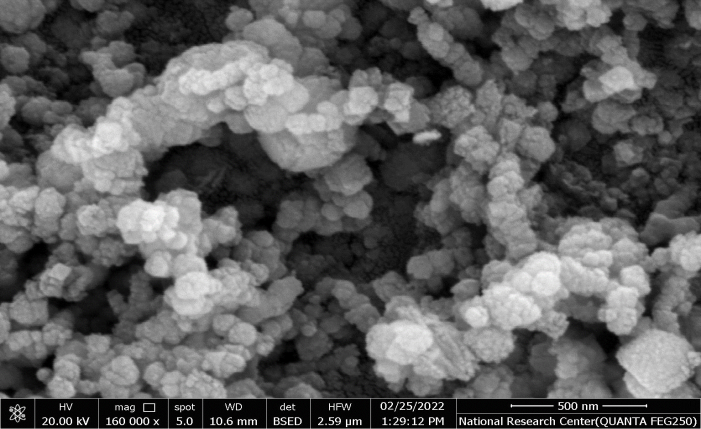** | **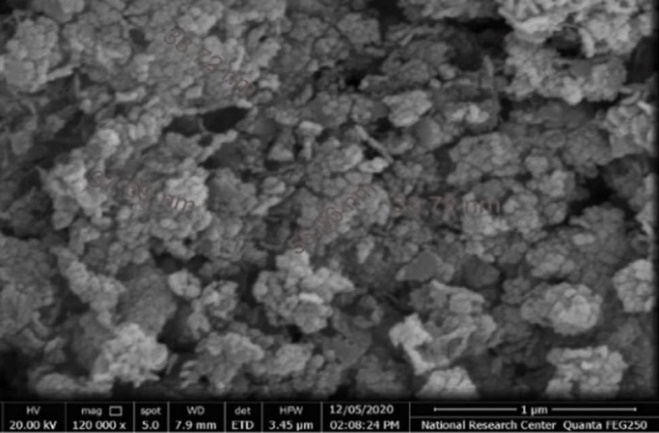** |
| --- | --- |

The SEM of (a) FeNPs-M and (b) FeNPs-T investigation

The summarizing particle size and elemental compositions of FeNPs-M and FeNPs-T

| Iron oxide nanoparticle | FeNPs-T | FeNPs-M |
| --- | --- | --- |
| Fe_3_O_4_ % | 40.86 | 46.62 |
| Particle size (nm) | 50.8 | 80.2 |


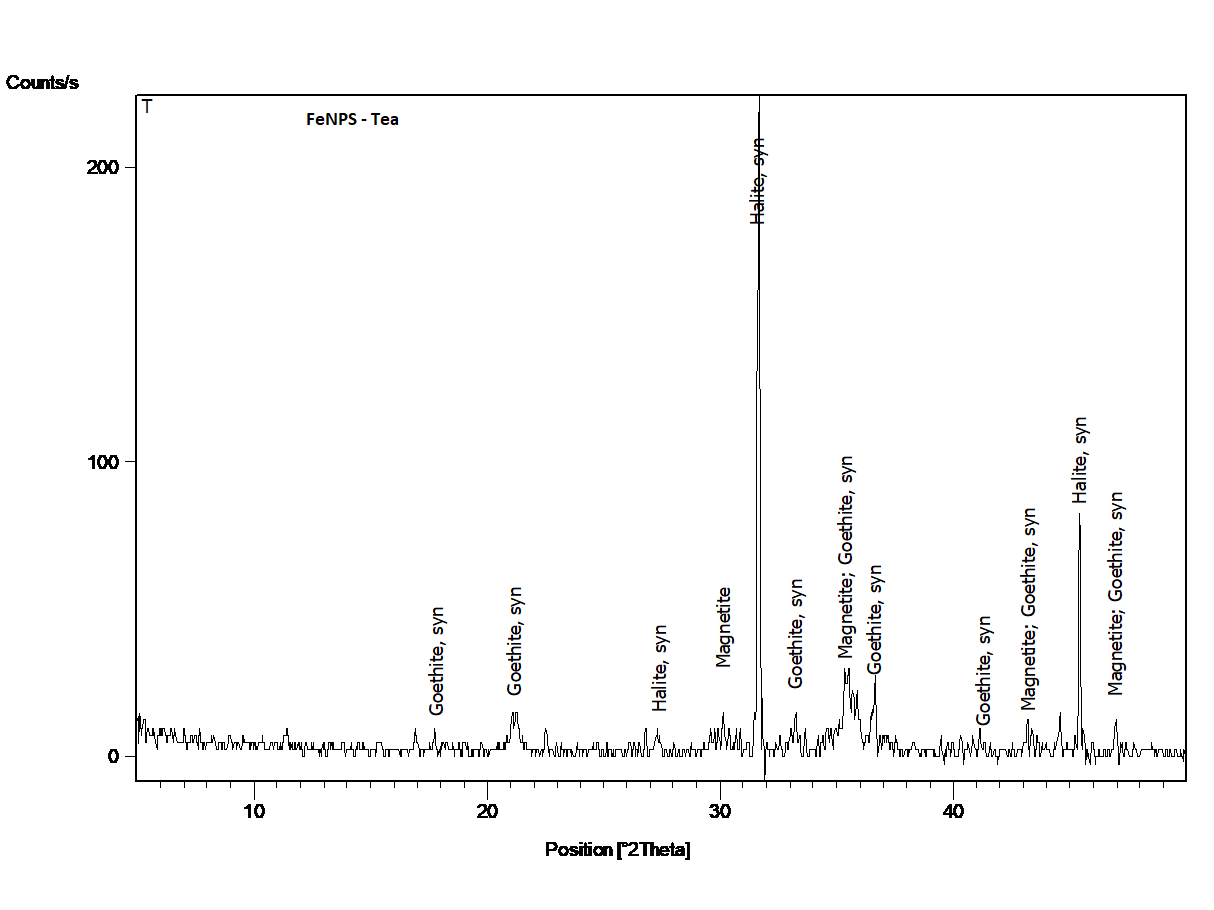

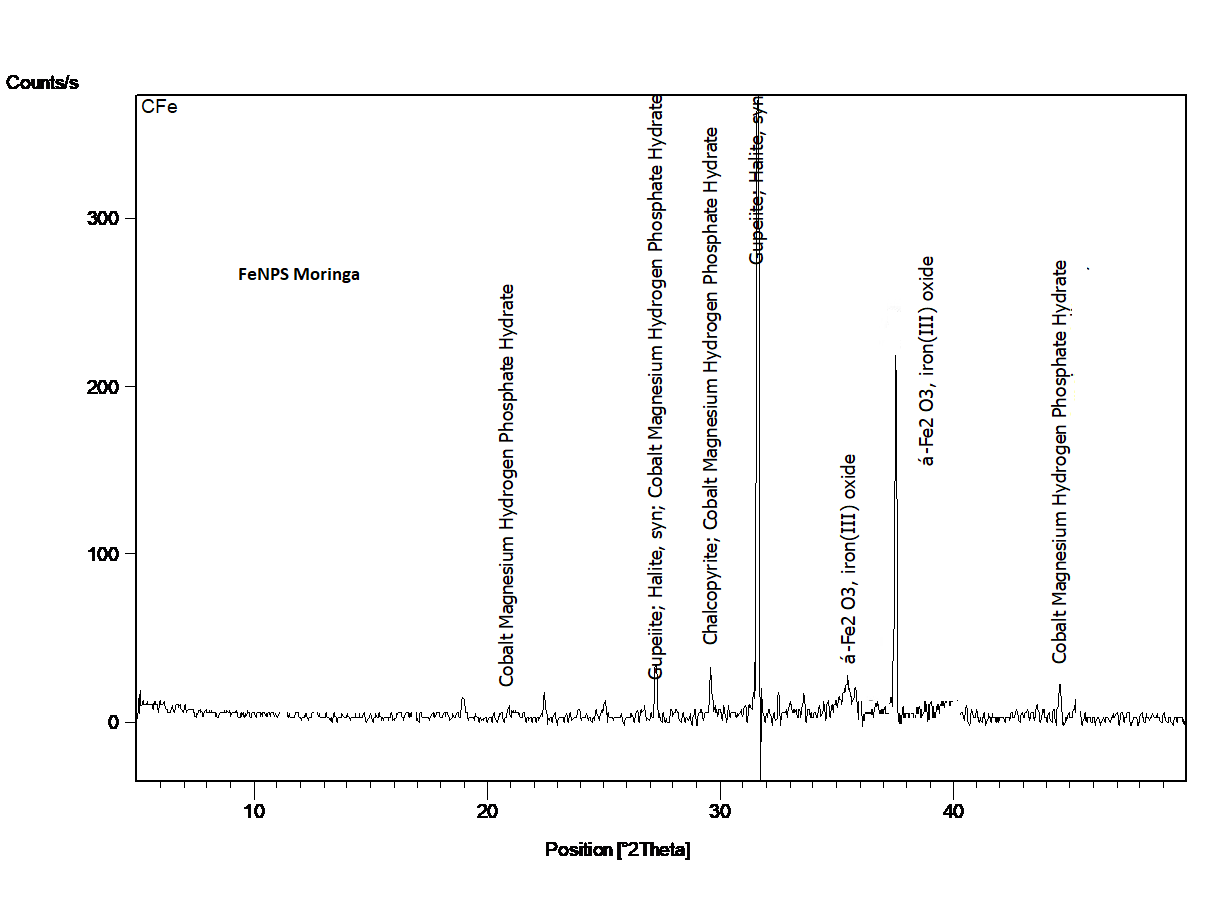


**The** XRD profile of (a) FeNPs-M and (b) FeNPs-T

The XRF of Fe_3_O_4_ nanoparticle FeNPs-M and FeNPs-T

| Iron oxide nanoparticle | Fe-metal | FeNPs-T | FeNPs-M |
| --- | --- | --- | --- |
| Fe_3_O_4_ % | 47.28 | 40.86 | 46.62 |
| Na_2_O % | 11.7 | 19.8 | 22.1 |
| MnO % | 0.43 | 0.33 | 0.46 |
| CaO % | 0.09 | 0.47 | 0.27 |
| SiO_2_ % | 0.28 | 0.38 | 0.36 |
| Al_2_O_3_ % | 0.06 | 0.11 | 0.07 |
| K_2_O % | 0.00 | 0.03 | 0.07 |
| SO_3_^2-^ % | 0.11 | 0.13 | 0.00 |
| Cr_2_O_3_ % | 0.04 | 0.01 | 0.04 |
| MgO % | 0.00 | 0.07 | 0.00 |
| Cl^-^ % | 10.1 | 15.6 | 14.9 |
| LOI % | 29.9 | 22.2 | 15.1 |
| Total % | 99.99 | 99.99 | 99.99 |

The XRF of Fe_3_O_4_ nanoparticle FeNPs-M and FeNPs-T

| Iron oxide nanoparticle | Fe-metal | FeNPs-T | FeNPs-M |
| --- | --- | --- | --- |
| Fe_3_O_4_ % | 47.28 | 40.86 | 46.62 |
| Na_2_O % | 11.7 | 19.8 | 22.1 |
| MnO % | 0.43 | 0.33 | 0.46 |
| CaO % | 0.09 | 0.47 | 0.27 |
| SiO_2_ % | 0.28 | 0.38 | 0.36 |
| Al_2_O_3_ % | 0.06 | 0.11 | 0.07 |
| K_2_O % | 0.00 | 0.03 | 0.07 |
| SO_3_^2-^ % | 0.11 | 0.13 | 0.00 |
| Cr_2_O_3_ % | 0.04 | 0.01 | 0.04 |
| MgO % | 0.00 | 0.07 | 0.00 |
| Cl^-^ % | 10.1 | 15.6 | 14.9 |
| LOI % | 29.9 | 22.2 | 15.1 |
| Total % | 99.99 | 99.99 | 99.99 |


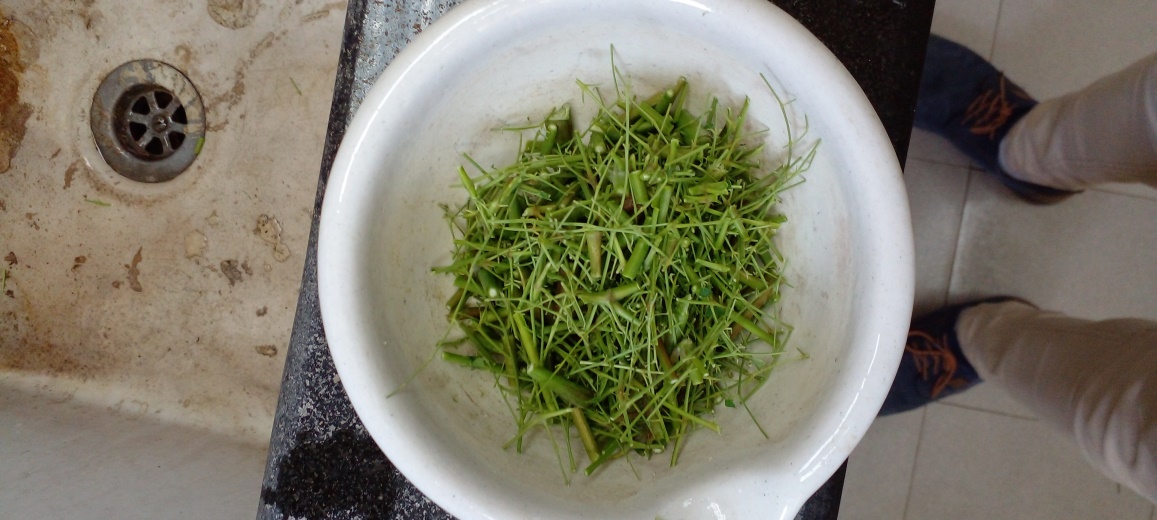

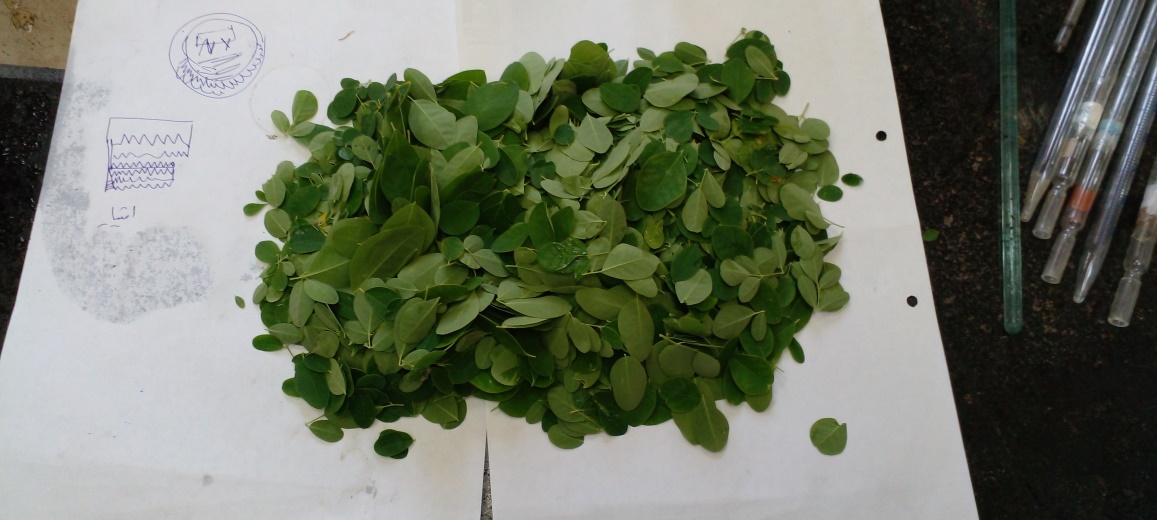

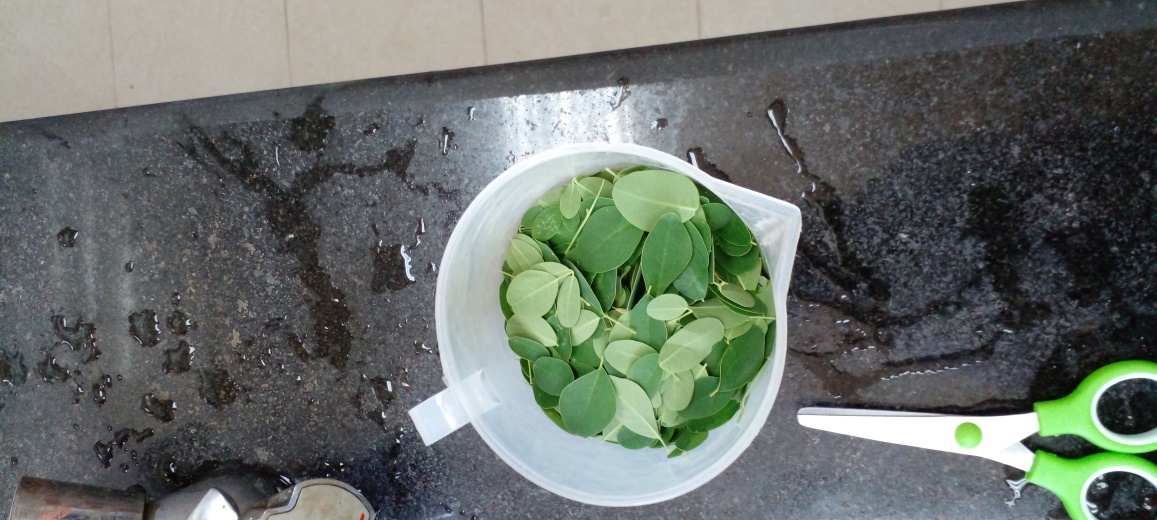

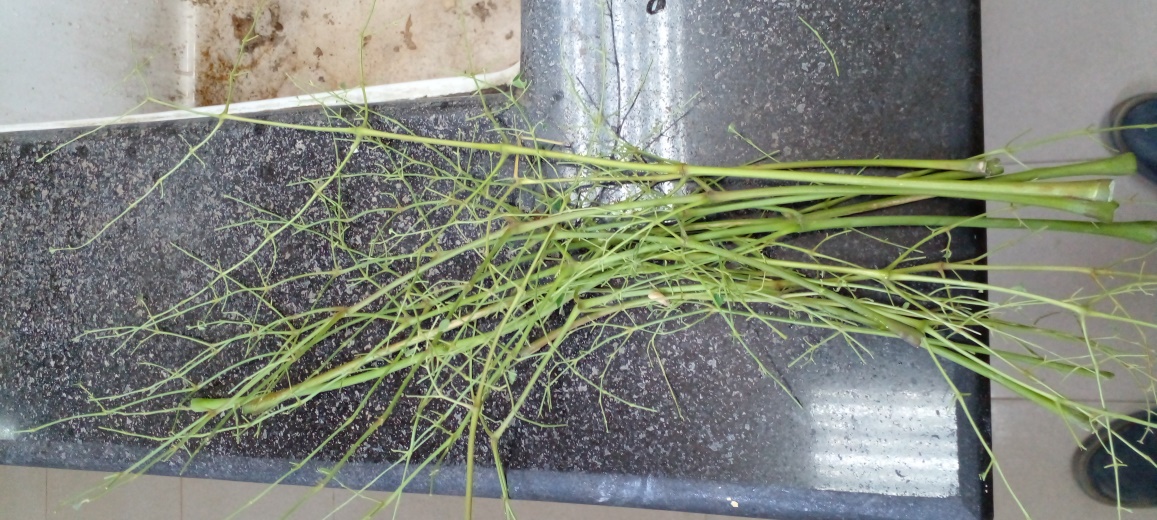

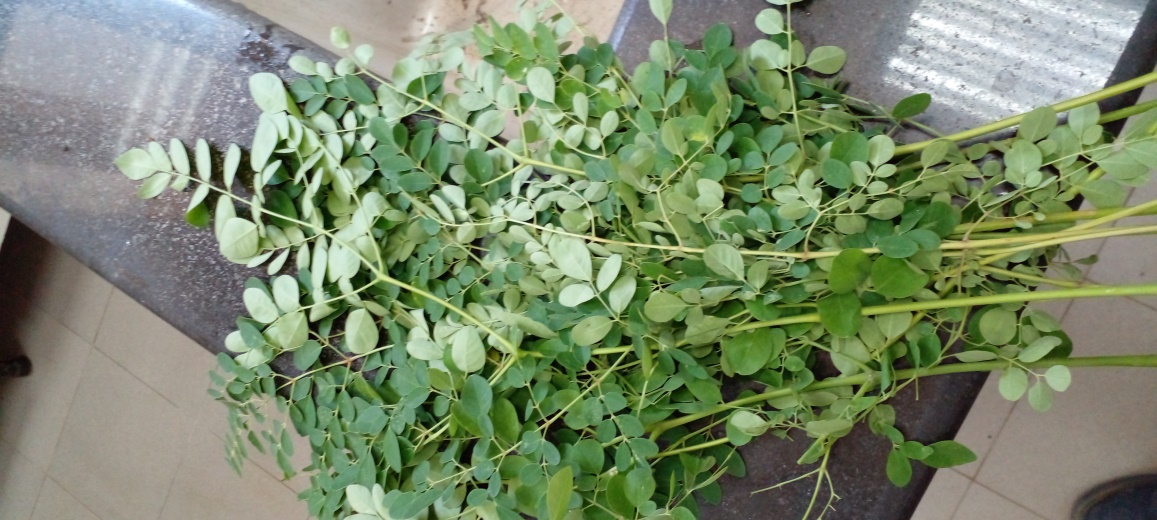

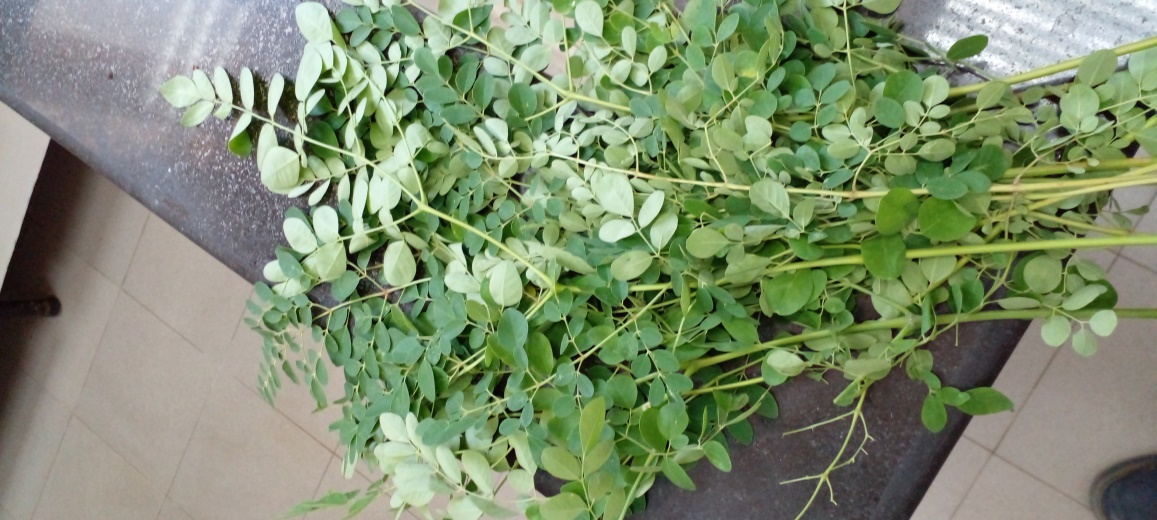

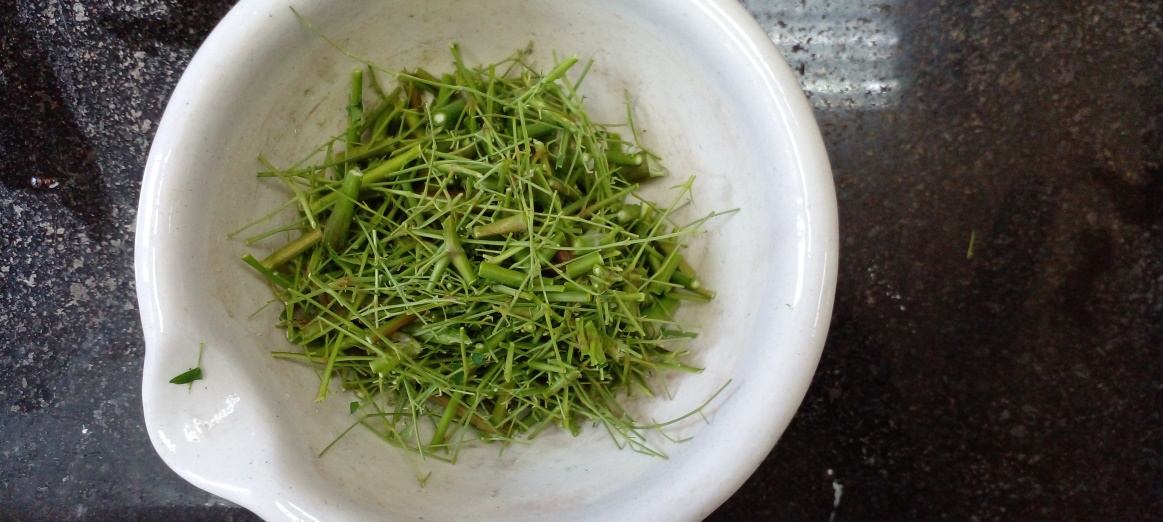


Moringa


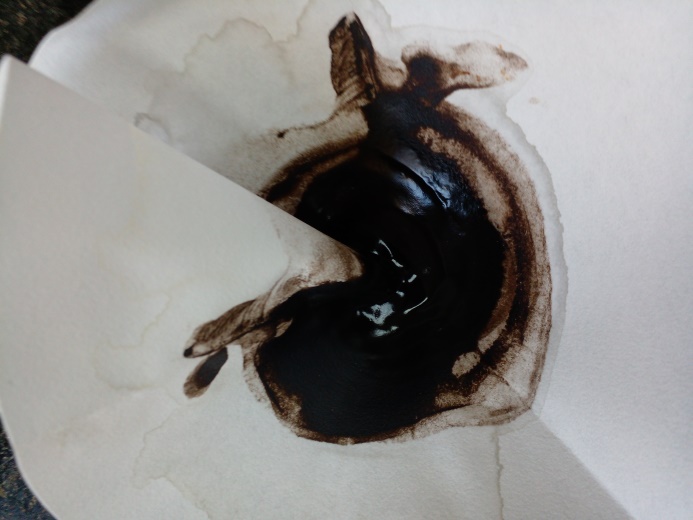

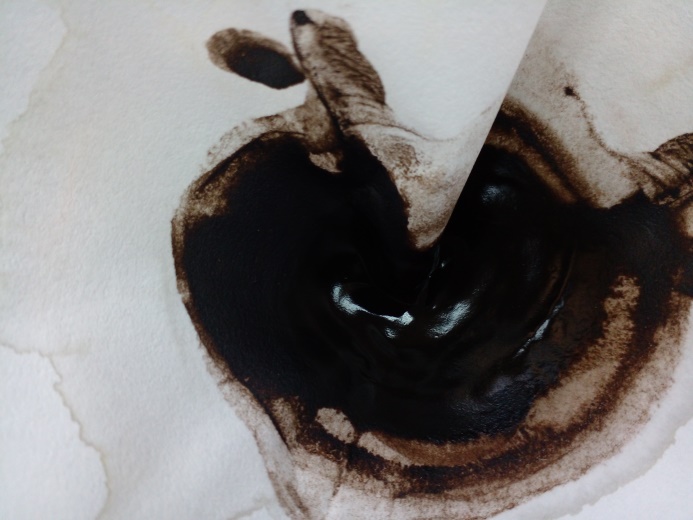


FeNPS


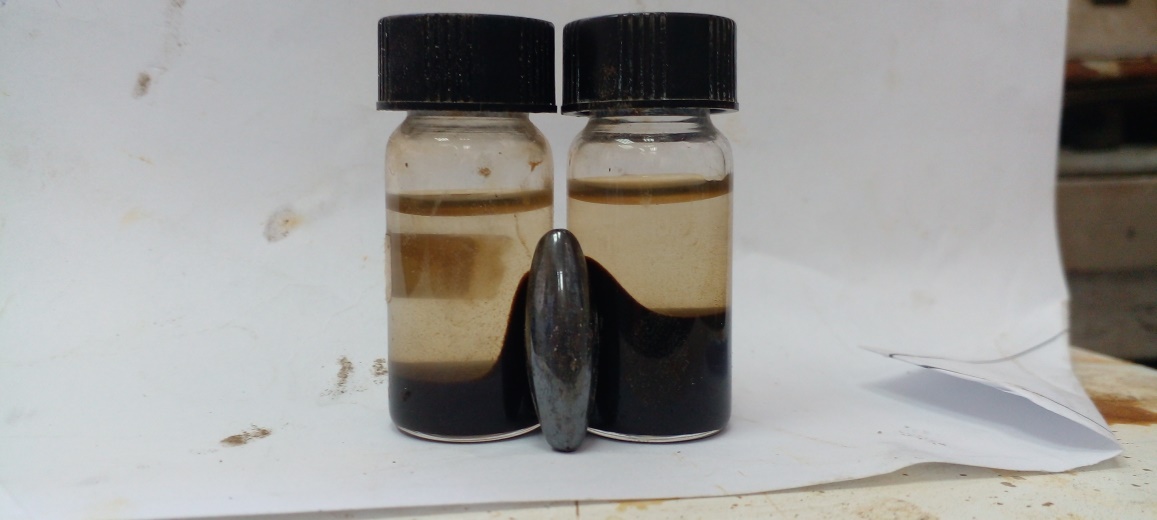

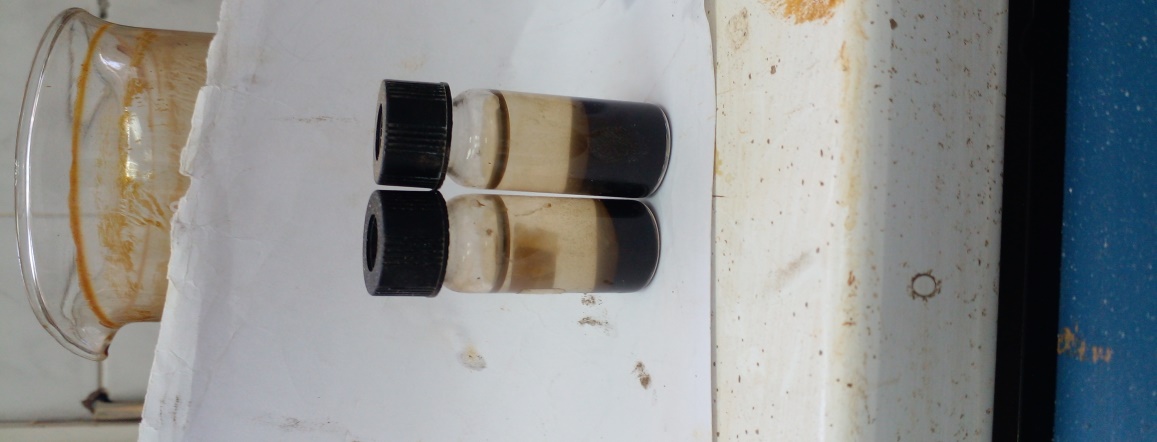

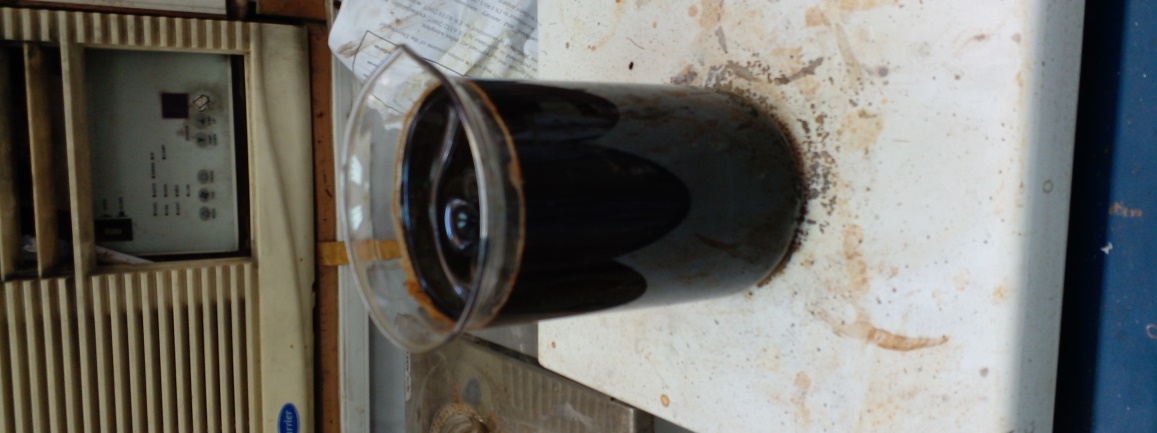

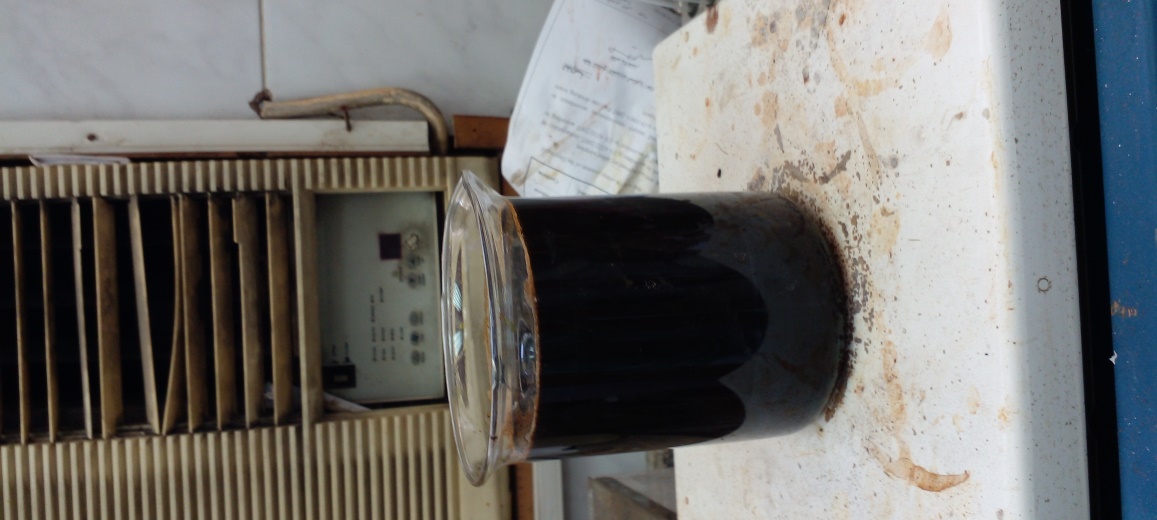

Supplement: S2 — (DOCX) [file pone.0329686.s002.docx]
